# Guideline for standardized approach in the treatment of the Mal de Debarquement syndrome

**DOI:** 10.3389/fneur.2024.1359116

**Published:** 2024-03-19

**Authors:** Catho Schoenmaekers, Steven Jillings, Chloë De Laet, Andrzej Zarowski, Floris L. Wuyts

**Affiliations:** ^1^Lab for Equilibrium Investigations and Aerospace, University of Antwerp, Wilrijk, Belgium; ^2^European Institute for ORL-HNS, Sint-Augustinus Hospital, Wilrijk, Belgium

**Keywords:** Mal de Debarquement syndrome, perception of self-motion, neuro-vestibular disorder, readaptation, guideline for MdDS treatment

## Abstract

**Introduction:**

Mal de Debarquement Syndrome (MdDS) is a debilitating neuro-otological disorder. Patients experience almost continuously a perception of self-motion. This syndrome can be motion-triggered (MT-MdDS), such as on a boat, or occur spontaneously or have other triggers (SO-MdDS) in the absence of such motion. Because the pathophysiological mechanism is unknown, treatment options and symptom management strategies are limited. One available treatment protocol involves a readaptation of the vestibular ocular reflex (VOR). This study assesses the effectiveness of vestibulo-ocular reflex (VOR) readaptation in 131 consecutive patients with a fixed protocol.

**Methods:**

We administered 131 treatments involving optokinetic stimulation (OKS) paired with a fixed head roll at 0.167 Hz over two to five consecutive days. Each day, four-minute treatment blocks were scheduled twice in the morning and afternoon. Treatment effectiveness was evaluated through questionnaires and posturography.

**Results:**

We observed significant improvements in the visual analog scale (VAS), MdDS symptom questionnaire, and posturography measures from pre- to post-treatment. No significant differences were found in outcome variables between MT- and SO-MdDS onsets.

**Conclusion:**

Symptoms improved subjectively and objectively in patients’ post-treatment. The overall success rate was 64.1%, with no significant difference between MT (64.2%) and SO (63.3%). This study supports the conclusion that VOR readaptation treatment provides relief for two-thirds of MdDS patients, irrespective of the onset type. Based on consistency in the findings, we propose a standardized method for treatment of MdDS based on the OKS with head roll paradigm.

## 1 Introduction

### 1.1 Mal de Debarquement syndrome: a debilitating condition

Mal de Debarquement (MdD), often referred to as “sea legs,” is a poorly understood neuro-otological condition characterized by the atypical sensation of non-spinning vertigo or perception of self-motion, especially when an individual is not moving at all. The sense of movement is often described as rocking (front-back motion), swaying (left–right motion), and/or bobbing (up-down motion) that is continuously present or for most of the day. Patients can also experience a gravitational pull, which is the feeling of being pulled to one side, or experience a ‘trampoline walk’ during locomotion. As soon as the patient moves passively, e.g., being in a car, the perception of self-motion disappears, but it returns after the motion stops. When this condition persists for longer than a month after the onset, it is denoted as Mal de Debarquement Syndrome (MdDS) (1). Patients suffering from MdDS frequently describe their experiences as to being on a ship or feeling unsteady on their feet. These symptoms may aggravate by various factors, such as exposure to busy environments, visual stimuli, or fatigue, making daily life exceedingly challenging for those affected. MdDS is often triggered after being exposed to passive motion, e.g., after a journey on a boat, car, train, or plane, but can also be triggered in the absence of such a passive motion event. This led to a preliminary classification of MdDS into a motion-triggered (MT-MdDS) variant and a ‘non motion-triggered’ variant, further denoted as spontaneous or other onset MdDS (SO-MdDS) (2).

To this day, MdDS is categorized as a rare disorder, although this classification may not accurately reflect the prevalence of the condition. This discrepancy arises from the significant number of individuals who remain undiagnosed due to the challenges of accurately identifying the syndrome (3). The prevalence of this condition has only been assessed in one study to date, where it was estimated to have an occurrence rate of 1.3 to 3% (similar to Menières disease) in a neuro-otological clinic (1). On average, MdDS patients undergo 19 consultations with healthcare professionals before receiving a correct diagnosis, where this diagnostic process can last several years. In a retrospective study, researchers reported that MdDS imposes economic burdens for the patients and the health care system of roughly 2,997$ (US dollars) per patient (3).

Only in December 2020, the first consensus paper regarding the diagnosis and classification of MdDS by the International Bárány Society was published (2). This inevitably yields that most healthcare professionals are not aware of MdDS. Consequently, treatment options and symptom management strategies are poor and inadequate. The absence of proper recognition and ineffective symptom management inevitably takes a toll on the patient’s mental well-being, lifestyle, and, consequently, their overall quality of life (QoL). It affects their employability, social life, and will to live (2, 4). While there exists a range of treatment options, including pharmacological interventions, neuromodulation, and vestibulo-ocular reflex (VOR) rehabilitation, their accessibility is limited, or they primarily address secondary symptoms rather than resolving the underlying issue (5). Therefore, adequate treatment options capable of diminishing MdDS symptoms are of great importance.

### 1.2 The cause of the Mal de Debarquement syndrome

While vertigo typically results from a peripheral malfunction of the vestibular organ, standard vestibular tests do not reveal any abnormalities in MdDS patients. Consequently, it is hypothesized that MdDS originates within the central nervous system (CNS), related to a maladaptation to passive motion (6). Neuroimaging studies have already revealed that MdDS patients show altered functional connectivity, metabolic activity, and gray matter volume compared to healthy controls (6, 7). It is hypothesized that this syndrome originates from a maladaptation of the velocity storage mechanism, which plays a role in the VOR pathways (8).

Previous experiments in monkeys, four rhesus and one cynomolgus monkeys, provided more insight into the cause of MdDS (9). During these experiments, monkeys were rolled around a naso-occipital axis for several hours from side-to-side while being rotated in darkness on an earth vertical axis. When the animals were rolled around the naso-occipital axis, only an ocular counter-roll was present. Subsequently, when the monkeys were adapted to the rolling, they also developed horizontal and vertical nystagmus. Besides that, the horizontal nystagmus velocity declined and when the monkeys were rolled side to side the vertical nystagmus shifted with an upward or downward slow phase depending on which side they were rolled. The observed changes in eye movements pointed to a maladaptation in the VOR. Notably, this maladaptive response only occurred in monkeys with long VOR time constants, suggesting its dependence on the central integrative mechanism of the vestibular system, such as the velocity storage mechanism (8, 10, 11).

In a more recent study, three rabbits were exposed to an unnatural repetitive motion for 2 h that involved intricate combinations of roll, pitch, and yaw movements in a head-based reference frame (12). The head-based frame was used to describe the six degrees of freedom, translation and rotation around the x, y, and z-axis with the rabbit’s head as reference. Eye movements were recorded before, during, and up to several days after the conditioning process. The results revealed that, following conditioning, an unusual ocular drift occurred in the yaw plane during rolling maneuvres, moving in the opposite direction to the nystagmus observed during conditioning. This finding suggests a maladaptive alteration and emphasizes the value of this rabbit model in MdDS research (12).

A similar experiment was performed in seven male volunteers in a rotating room that revolved for on average 5 times a minute (5.4 revs/min) for 64 h (13). A nystagmus was observed more than an hour after the rotational motion ceased. Taking both subhuman and human data together, it is hypothesized that MdDS arises from a maladaptation of the VOR to a sustained complex movement, specifically driven by the velocity storage mechanism. In line of this, MT-MdDS patients undergo extended exposure to passive motion, such as on a boat, similar to the laboratory situations of the studies described above. The velocity storage mechanism likely uses the boat’s movement to counteract this motion as an adaptation mechanism, preserving adequate balance on the boat. Upon disembarking, a readaptation must occur to account for a stationary environment. Failure of such readaptation might then cause the sensation of self-motion during standstill on solid ground, as the velocity storage mechanism continues to align with the boat’s motion. It is important to note that while this hypothesis applies to MT-MdDS patients, those with SO-MdDS exhibit similar symptoms, though the exact mechanism of onset remains even more elusive.

Based on the VOR maladaptation hypothesis, a previous study by Dai and colleagues indicated that a treatment protocol based upon the re-adaptation of the VOR relieves the perception of self-motion (8). They treated a total of 24 MT-MdDS patients by rolling their head side-to-side while watching a rotating full-field visual stimulus. This treatment engages the visual pathways which transmit their input to the velocity storage in the vestibular nuclei, and further to the inferior olivary nucleus and cerebellum (10). Out of the 24 MT-MdDS patients, 16 showed complete or substantial recovery, six were initially better, and only one did not respond to the treatment. They showed that the readaptation of the VOR has led to improve the perception of self-motion by 70%. Based on previous studies, we aimed to investigate if the VOR maladaptation could be reversed by using optokinetic stimulation in combination with a head roll at 0.167 Hz (8, 14). Hereto, we report the results of a large group of consecutive patients treated with this fixed protocol in our clinical setting in Antwerp. We aimed to examine the outcome of the treatment protocol, based on subjective and objective outcome measures, to examine the difference between both onsets, patients who were treated once or multiple times, and demographic information such as age and sex.

## 2 Materials and methods

### 2.1 Subjects

Between August of 2019 and December of 2021, we treated a total of 101 patients, who consecutively visited the ENT department at the St Augustinus hospital in Antwerp. Some individuals had multiple treatments during this period, hence the total number of treatments was 131. Nineteen patients came back for a second treatment session, six for a third, and five for a fourth. From the 101 individual patients, 81 are female of on average (SD) 45 (15) years old and between 18 and 79 years of age, and 20 male of on average (SD) 39 (12) years old and between 24 and 61 years of age. Patients had had complaints for a median (median absolute deviation (MAD)) of 3.7 (1.78) years and between 1 month and 21 years, before they received the OKS treatment.

For MT-MdDS patients, the complaints started after a trip with a boat, car, bus, plane, or a train. Other, more uncommon triggers were after using a hometrainer, and for another patient after surfing. The triggers for the SO-MdDS patients were: Menière’s disease, benign paroxysmal positional vertigo (BPPV), acute unilateral vestibulopathy (neuritis), vestibular paroxysmia, giving birth, immunotherapy, treatment by chiropractor, quitting or changing contraceptive medication, or virtual reality. Some patients (32%) did not recall a specific event associated with the onset of their symptoms, they are therefore also categorized as SO-MdDS patients.

Prior to undergoing treatment, MdDS patients received their diagnosis by MdDS expert FW, during a consultation with an Ear, Nose, and Throat (ENT) physician at the European Institute for Otorhinolaryngology, located at the Sint-Augustinus Hospital in Wilrijk, Belgium. Patients were assessed using the SO STONED anamnesis, which stands for Symptoms, Often, Since, Trigger, Otology, Neurology, Evolution, and Duration (15). The comprehensive assessment across these dimensions contributed to a distinctive profile of the specific vestibular disorder (15). This retrospective study was approved by the ethical committee of the Gasthuis Zusters Antwerpen (GZA) hospitals in accordance with the Declaration of Helsinki.

### 2.2 Treatment

Patients were treated based on the optokinetic stimulation (OKS) protocol of Dr. Dai (8). We used a more standardized protocol for every patient similar to the one published earlier by our group (14). The treatment involves a combination of head-roll movements while exposing the patient to a moving stripe pattern, i.e., the OKS stripes. The velocity of OKS was 10° per second and the clinician simultaneously oscillated the patient’s head manually within the frontal plane (left to right shoulder) with a range of ±20° and at a strict frequency of 0.167 Hz. Patients were instructed to passively track the moving stripes while maintaining their gaze forward. The frequency of 0.167 Hz was chosen as it corresponds to the resonance frequency of the vestibular system (16). The clinician rolled the patient’s head guided by an auditory cue, in the form of a music piece with an ascending and descending melody of a 6-s period.

**Figure 1:**
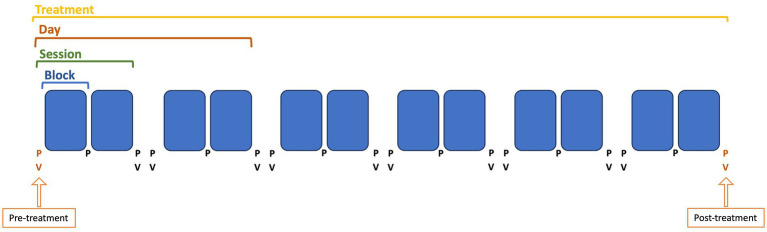
Schematic overview about how a treatment is structured. Pre-treatment is defined as the first posturography (P) and visual analog scale score (V), before the first treatment block. Post-treatment is defined as the last posturography (P) and visual analog scale score (V), after the last treatment block. One treatment block is 4 min, therefore, the latency between a pre- and post-treatment block for the posturography is 4 min each time. Regarding the VAS score, taken before and after two treatment blocks, the latency is 8 min between two VAS score measurements.

All patients underwent 2 to 5 days of OKS treatment. Each day, a treatment session was scheduled in the morning and afternoon, where each session consisted of two blocks of 4 min of OKS treatment (Figure 1). On the first day of treatment, the direction of the OKS stripes was determined using the Fukuda stepping test and/or the patient’s subjective perception of the internal oscillation. The direction of the stripes could be toward the left, right, upwards, or downwards. During the Fukuda stepping test, patients were instructed to close their eyes and walk in place with their arms extended forward for 45 s. Based on the patient’s direction of rotation during this stepping test, the administered OKS stripes moved in the opposite direction. For instance, if the patient exhibited a deviation to the left, the stripes would move toward the right. If the deviation was more in a forward motion, stripes moving downward were used. If the Fukuda stepping test was negative, with no clear deviation, the direction of the stripes was determined based on the patient’s description of their perceived motion or the direction of gravitational pull. For sway sensations, stripes moving toward the left or right were mostly used. For rocking and bobbing sensations, stripes moving downwards or upwards were mostly used.

### 2.3 Outcome measures

To quantify the treatment’s efficacy, we used a combination of subjective and objective measurements. For subjective assessment, two distinct questionnaires were used. On the treatment days, MdDS patients were asked to rate the intensity of their symptoms by putting a vertical dash on a visual analog scale (VAS) of 10 cm long, with 0 indicating no disturbance and 10 cm corresponds with maximum perception of self-motion. Patients used this VAS to express their sense of instability both before and after each treatment session. Additionally, patients completed a dedicated questionnaire focused on MdDS symptoms including fatigue, headache, eye strain, difficulties in focusing, increased salivation, sweating, nausea, brain fog (lack of focus and mental clarity), blurry vision, dizziness with eyes open, dizziness with eyes closed, vertigo, problems with orientation, and uncomfortable feeling in the stomach. These symptoms were likewise assessed by means of a VAS. The patients filled in this questionnaire on the first day (before the first session) and on the last day (after the last session) of the treatment.

For objective assessment, we used posturography by means of a Wii balance board (Wii BALANCE BOARD. RVL-021. Nintendo Co., Ltd. 11–1 Kamitoba-Hokotate-cho Minami-ku. Kyoto, 601–8,501 Japan), before, after, and in between each treatment block (Figure 1). Posturography is a functional evaluation of postural control and stability, which is ensured by the interaction between different sensory systems (i.e., visual, vestibular, and somatosensory systems). The Wii balance board shows promising results regarding posturography analysis in vestibular patients (17). The balance board measures the subject’s frequency and speed of movement by means of four pressure sensors, located under the support points of the board. In normal subjects, postural sway is also present, with values ranging from 3.17 mm for lateral sway amplitude to 1.44 mm for sagittal/lateral sway ratio (18). In MdDS patients, increased sway and sway ratios are visible due to the oscillatory perceptions of self-motion. The sway will cause patients to redistribute their center of pressure more toward one side of the board and, therefore, the pressure sensors on that side of the board will register an increase in weight. A previous study performed by our team included posturography data of 20 healthy volunteers who were evaluated at 2 different moments, to assess the reliability of the outcome variables. Based on these data, we have concluded that the posturography measures are significantly increased in MdDS patients, as well that the improvement observed in patients after treatment, is significantly larger than the within-subject variability of the healthy controls (14). For this research, a static posturography was taken before and after each treatment block. Patients stood on the Wii-balance board with the arms crossed in front of the chest and with eyes closed for 1 min. The data was analyzed with a program based on the Colorado University Wii Balance Board code developed at the Neuromechanics Laboratory at Colorado University.^1^ After acquisition, the data was filtered using a Butterworth filter of fourth order and a cut-off frequency of 0.17 Hz (MATLAB—Release 2023a, developed by The MathWorks, Inc., Natick, Massachusetts, United States) (14). With a sampling rate of 100 Hz, the sensitivity of Wii Balance Board in detecting a balance dysfunction was determined to be 69.39%, the specificity 73.16% (19). Using this customized MatLab routine, we were able to extract the speed of movement and the total distance traveled. Additionally, the confidence ellipse area (CEA) is calculated with a 95% confidence interval (CI). The CEA is the area of the ellipse containing 95% of the posturography measurement. The MatLab routine additionally uses a discrete Fourier transformation (DFT) to generate a power spectrum displaying the frequency of movement, medio-laterally (ML) and antero-posteriorly (AP). The DFT extracts the spectrum of a finite-duration signal, i.e., it determines the frequency content of a time-domain signal. The area under the curve (AuC) of these power spectra, as a measure of energy content, is subsequently calculated in the ML and AP direction, denoted as AuC-ML and AuC-AP.

### 2.4 Statistical analysis

All statistical analyses for the outcome measurements were conducted in JMP^®^ (version Pro 16, SAS Institute Inc., Cary, NC, 1989–2001), with a significance threshold set at alpha = 0.05. To assess the overall impact of optokinetic stimulation from pre- to post-treatment, we employed four linear mixed models (LMMs), one for each outcome measure (VAS scores, CEA, AuC-ML, and AuC-AP), using a stepwise backward approach. This method initiates with a comprehensive model containing all relevant independent variables and interaction terms. Subsequently, at each step, non-significant variables and interaction terms are removed until only the significant ones remain in the model, resulting in an optimized model that best explains the data. The dependent variables in the models included VAS scores, CEA, AuC-ML, and AuC-AP. Independent variables included were timepoint (pre- and post-treatment), sex (male—female), onset type (MT–SO), symptom duration, age, and treatment number (a patient’s number of treatments, range 1–4). Patient number was incorporated as a random intercept in all models to address non-independence among observations from the same patient. Additionally, the random slope term patient_number*timepoint was introduced as a random effect if it significantly enhanced the model fit, determined through the Likelihood ratio test. To ensure the validity of the models, we examined the residuals for normality and homoscedasticity. Descriptive statistics were used to gain insights into the dataset and study population, including parameters such as age, sex, symptom duration, and onset type distribution. Chi-square tests were used to analyze the frequency of the direction of the optokinetic stimulation (OKS) stripes and sex. Pearson correlation tests were used to explore correlations between objective and subjective measurements, investigating if subjective improvement reported via VAS scores corresponded with changes in posturography measurements. Furthermore, we employed non-parametric Wilcoxon tests to identify significant differences in specific symptoms (e.g., brain fog, fatigue, headache) from pre- to post-treatment. A Mann–Whitney U test was applied to investigate whether onset type had an effect on the pre- to post-treatment difference in symptom severity. To account for multiple comparisons, we applied a Benjamini-Hochberg correction for the Wilcoxon and Mann–Whitney U tests using RStudio (version 1.2.1335).

## 3 Results

Since the VAS scores, the CEA, the AuC-ML, and the AuC-AP measurements were recorded on different occasions within the same patient, the modeling of changes was therefore carried out in a LMM framework. All estimates can be found in Table 1, and all reported results are provided with standard errors.

**Table 1:** Overview of the estimates and *p*-values of the different linear mixed models (LMM).

Fixed effects	Estimate (cm)	Std error (cm)	CI 95% (cm)	*p*-value
Model 1: the effect of optokinetic stimulation on the subjective improvement, visual analog scale (VAS score), in MdDS patients				
Intercept	4.38	0.29	[3.80; 4.97]	**<0.0001**
Treatment (first)	0.14	0.24	[−0.35; 0.63]	0.5743
Treatment (second)	−0.31	0.25	[−0.81; 0.20]	0.2265
Treatment (third)	0.14	0.33	[−0.55; 0.82]	0.6860
Timepoint (pre-treatment)	1.73	0.21	[1.31; 2.15]	**<0.0001**
Treatment (first)*Timepoint (pre-treatment)	−0.65	0.23	[−1.11; −0.19]	**0.0058**
Treatment (second)*Timepoint (pre-treatment)	−0.47	0.30	[−1.06; 0.12]	0.1159
Treatment (third)*Timepoint (pre-treatment)	0.72	0.43	[−0.13; 1.57]	0.0981
Model 2: the effect of optokinetic stimulation on the objective improvement, confidence ellipse area (CEA), in MdDS patients				
Intercept	19.24	4.75	[9.78; 28.70]	**0.0001**
Timepoint (pre-treatment)	7.06	2.95	[1.21; 12.91]	**0.0186**
Model 3: the effect of optokinetic stimulation on the subjective improvement, area under the curve for posterior–anterior movement (AuC-AP), in MdDS patients				
Intercept	2.12	0.42	[1.29; 2.94]	**<0.0001**
Timepoint (pre-treatment)	0.73	0.26	[0.21; 1.25]	**0.0063**
Model 4: the effect of optokinetic stimulation on the subjective improvement, area under the curve for medio-lateral movement (AuC-ML), in MdDS patients				
Intercept	0.99	0.23	[0.53; 1.44]	**<0.0001**
Timepoint (pre-treatment)	0.32	0.14	[0.05; 0.59]	**0.0198**

CI, confidence interval. Timepoint (post-treatment) was taken as the reference value. Treatment (fourth) was taken as the reference value.

### 3.1 Overall effect of optokinetic stimulation pre- to post-treatment

Our four models evaluated whether the VAS score, CEA, AuC-ML, and the AuC-AP measurements were dependent on age, treatment, symptom duration, sex, onset, and Timepoint.

**Figure 2:**
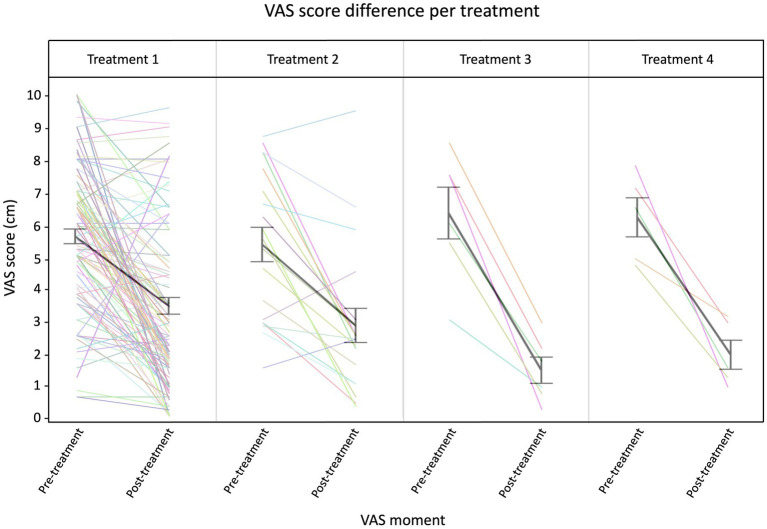
Raw traces of the VAS scores at Timepoint pre- and post-treatment. The thick black lines indicate an overall average per treatment (error bars ±1 SD). N treatment 1 = 101, N treatment 2 = 19, N treatment 3 = 6, and N treatment 4 = 5.

Our first model evaluated the VAS score for age, treatment number, symptom duration, sex, onset, and Timepoint. We observed a significant effect of the Timepoint (*p* < 0.0001), where the pre-treatment VAS score was higher (2.5 ± 0.2 cm) compared to post-treatment VAS score. Additionally, treatment number had a significant effect on the VAS score (Treatment*Timepoint, *p* 0.0326), meaning that VAS scores decreased more strongly when the number of treatments increased (Figure 2).

In addition, a *post hoc* analysis with Dunnett’s correction was performed to compare the VAS score between treatment numbers over pre- and post-treatment measurement timepoints. The average VAS score of treatment 1 pre-treatment was used as reference value. Variables that showed to be significantly different from this baseline value are the post-treatment scores of treatment 4 (*p* = 0.0003), treatment 3 (*p* < 0.0001), treatment 2 (*p* < 0.001), and treatment 1 (*p* < 0.0001).

**Figure 3:**
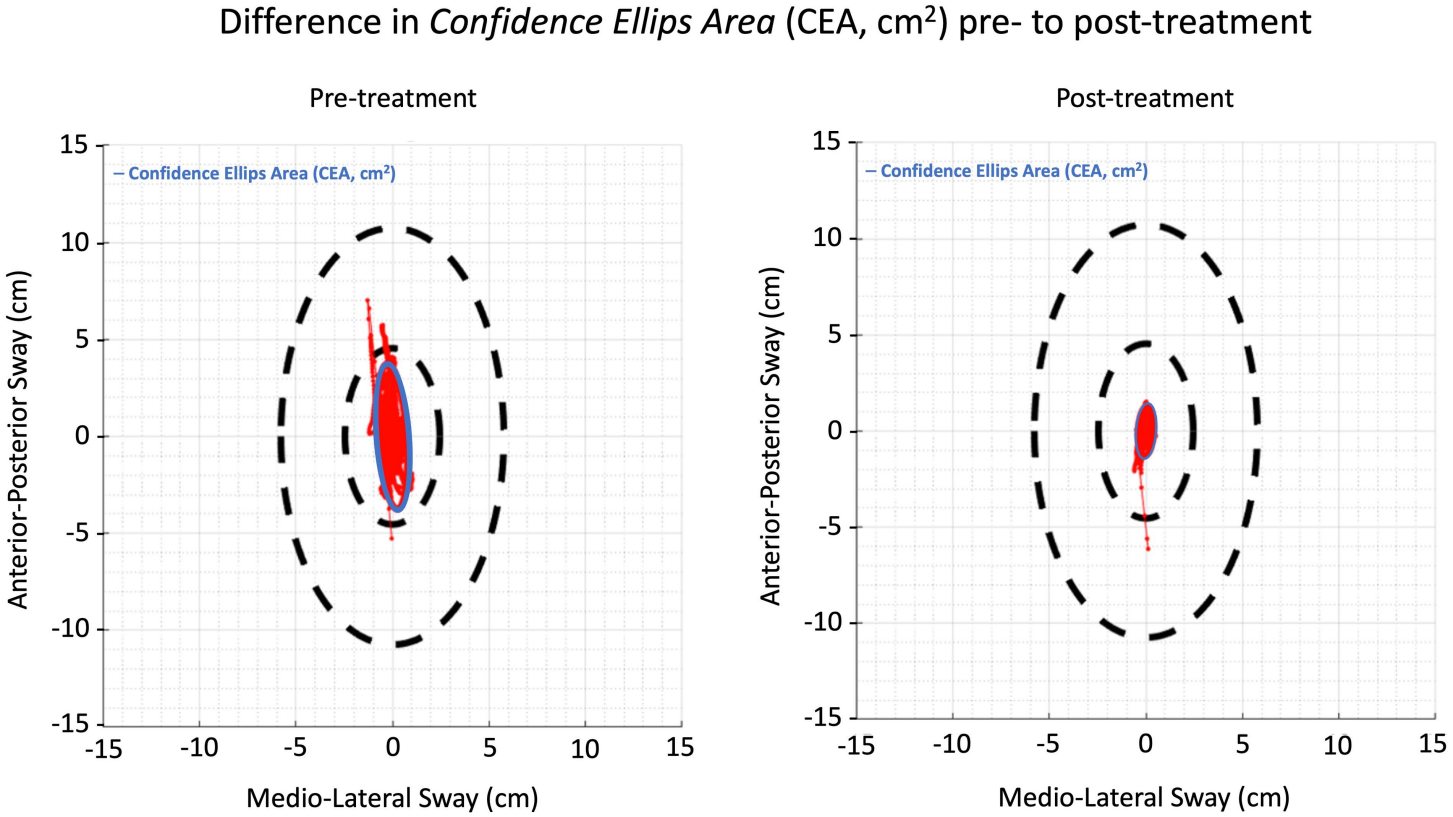
Illustration of the confidence ellipse area pre- to post-treatment in one patient.

Subsequently, three additional LMM’s were used to evaluate whether the three posturography outcome variables, CEA, AuC-AP, and AuC-ML were dependent on age, treatment (first until fourth treatment), symptom duration, sex, onset, and Timepoint. First, there was a significant effect of Timepoint (*p* = 0.0186) on the CEA. The average CEA pre-treatment was 26.3 cm^2^ ± 5.6 cm^2^, which was 14.1 cm^2^ ± 5.9 cm^2^ higher compared to the average CEA post-treatment of 12.2 cm^2^ ± 5.6 cm^2^ (Figure 3).

**Figure 4:**
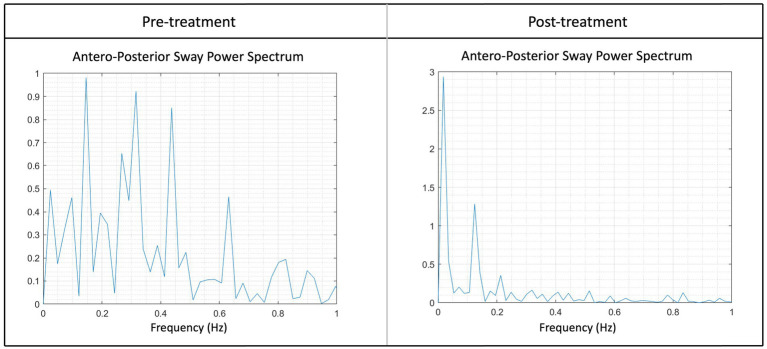
Illustration of the sway power spectrum pre- and post-treatment of the anterior–posterior sway (AuC-AP) for one patient. Higher peaks suggest increased rocking at a particular frequency, for instance, the peak at pre-treatment between 0.2–0.4 Hz and 0.4–0.5 Hz was notably higher. Following the treatment, a noticeable reduction in peaks at higher frequencies could be observed, indicating an enhanced stability post-treatment. Peaks within the frequency range of 0–0.2 Hz are considered typical and reflect a normal state. Note in both graphs the peak around 0.167 Hz.

The LMM with the AuC-AP as independent variable showed a significant effect of the Timepoint (*p* = 0.0063). The average AuC-AP pre-treatment was 2.9 cm^2^ ± 0.5 cm^2^, which was 1.5 cm^2^ ± 0.6 cm^2^ higher compared to the average AuC-AP post-treatment of 1.4 cm^2^ ± 0.5 cm^2^ (Figure 4).

**Figure 5:**
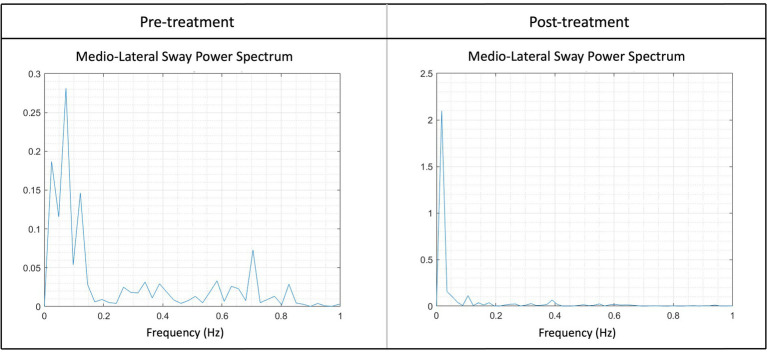
Illustration of the sway power spectrum pre- and post-treatment of the medio-lateral sway (AuC-ML) for one patient. Higher peaks suggest increased swaying at a particular frequency, for instance, the peak at pre-treatment between 0.6 and 0.8 Hz was notably higher. Following the treatment, a noticeable reduction in peaks at higher frequencies could be observed, indicating an enhanced stability post-treatment. Peaks within the frequency range of 0–0.2 Hz are considered typical and reflect a normal state.

Lastly, there was a significant effect of Timepoint (*p* = 0.0198) on the AuC-ML. The average AuC-ML pre-treatment was 1.1 cm^2^ ± 0.2 cm^2^, which was 0.6 cm^2^ ± 0.3 cm^2^ higher compared to the average AuC-ML post-treatment of 0.4 cm^2^ ± 0.3 cm^2^ (Figure 5).

In addition, we analyzed the correlation between the subjective and objective measurements using the Pearson correlation (Table 2). We showed a significant correlation between all measurements, namely the VAS score, CEA, AuC-ML, and AuC-AP.

**Table 2:** Results of the Pearson correlation tests between the three posturography outcome measures (CEA, AuC-ML, and AuC-AP) and VAS score.

Variable	By variable	Correlation coefficient (r)	Lower 95%	Upper 95%	*p*-value
CEA	VAS score	0.1797	0.04	0.31	**0.0119**
AuC-ML	VAS score	0.1937	0.05	0.33	**0.0067**
AuC-AP	VAS score	0.1742	0.03	0.31	**0.0149**
AuC-AP	AuC-ML	0.8620	0.82	0.89	**<0.0001**

Bold values were used to indicate significance.

**Figure 6:**
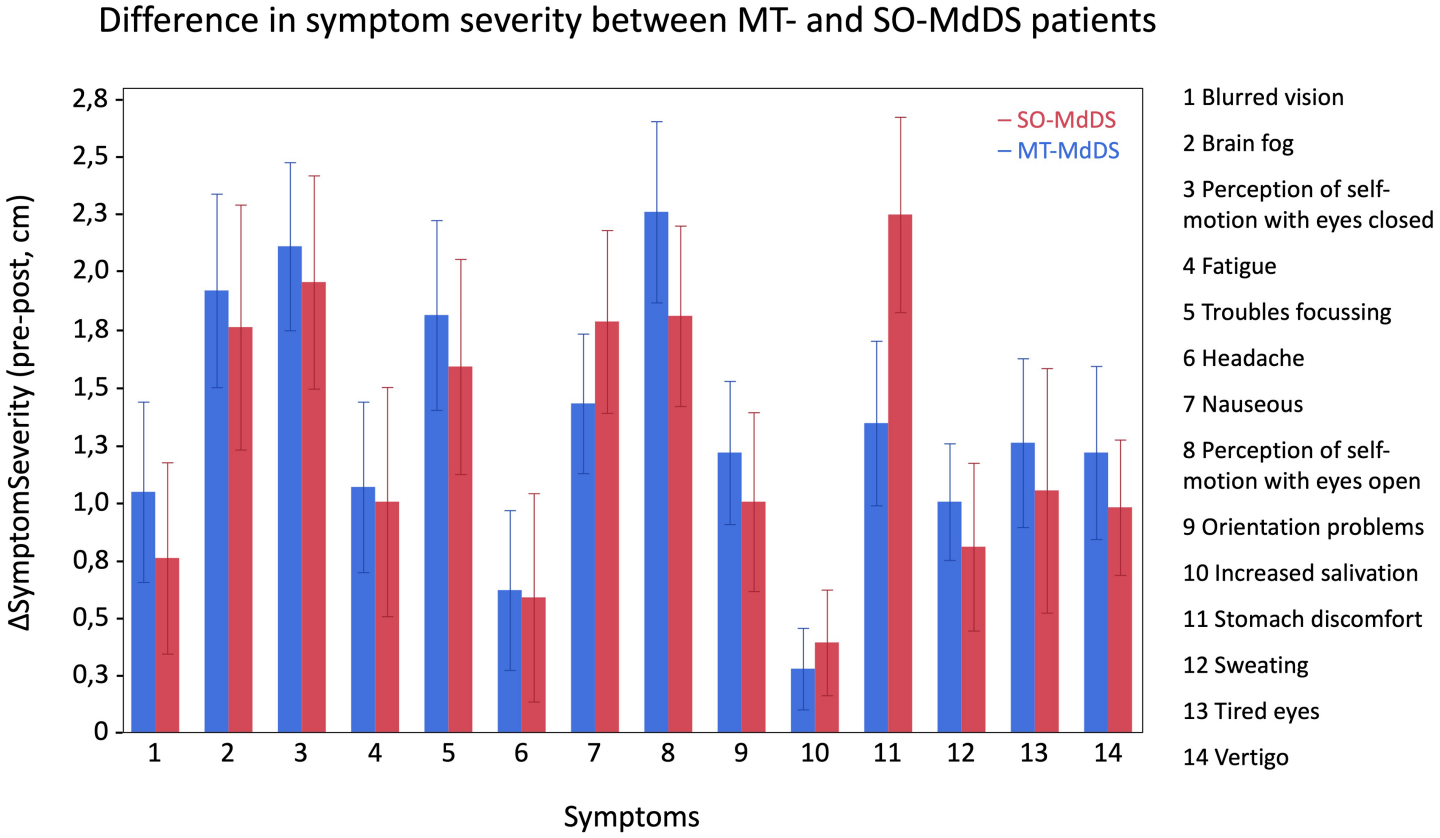
Overview of difference in symptom severity (ΔSymptomSeverity, pre-post) between MT- and SO-patients for each questioned symptom. The graphs indicates that both onsets show a similar improvement in symptom severity. A positive ΔSymptomSeverity indicates improvement in symptoms.

Since MdDS patients show a broad spectrum of symptoms we used a symptom specific questionnaire to investigate what the effect was of the OKS treatment. Using a Wilcoxon test we found significant improvements for all 14 questioned symptoms post-treatment (Table 3). Additionally, by using a Mann–Whitney U test (Table 4), the onset type did not influence the improvement of symptoms (Figure 6).

**Table 3:** Results of the Wilcoxon signed rank tests to evaluate changes in subjective experience after treatment compared to before (Δ).

Domain	Test statistics S	*p*-value	Adjusted *p*-value
Fatigue	−880.50	**0.0002**	**0.0003**
Headache	−604.50	**0.0106**	**0.0114**
Tired eyes	−769.50	**0.0005**	**0.0006**
Troubles focusing	−1208.00	**<0.0001**	**<0.0001**
Increased salivation	−410.00	**0.0256**	**0.0256**
Sweating	−1053.00	**<0.0001**	**<0.0001**
Nauseous	−1365.00	**<0.0001**	**<0.0001**
Brain fog	−1382.50	**<0.0001**	**<0.0001**
Blurred vision	−737.50	**0.0017**	**0.0020**
Perception of self-motion with eyes open	−1536.00	**<0.0001**	**<0.0001**
Perception of self-motion with eyes closed	−1450.00	**<0.0001**	**<0.0001**
Vertigo	−1221.00	**<0.0001**	**<0.0001**
Orientation problems	−1050.50	**<0.0001**	**<0.0001**
Stomach discomfort	−1381.00	**<0.0001**	**<0.0001**

Subjective experience was assessed in 14 domains by means of a VAS. Adjusted *p*-Values are calculated according to the Benjamini-Hochberg correction for multiple comparison.

Bold values were used to indicate significance.

**Table 4:** Results of the Mann–Whitney U tests to evaluate whether changes in subjective experience after treatment compared to before (Δ) was different between MT- and SO-MdDS patients.

Domain	Mann–Whitney U	Z	*p-*value	Adjusted *p*-value
Fatigue	963.00	−0.004	0.997	0.997
Headache	947.00	−0.141	0.888	0.956
Tired eyes	921.50	−0.354	0.723	0.956
Troubles focusing	946.50	−0.144	0.886	0.956
Increased salivation	900.50	−0.705	0.481	0.956
Sweating	816.00	−1.292	0.196	0.956
Nauseous	932.50	−0.267	0.789	0.956
Brain fog	894.00	−0.586	0.558	0.956
Blurred vision	927.50	−0.307	0.758	0.956
Perception of self-motion with eyes open	925.50	−0.321	0.748	0.956
Perception of self-motion with eyes closed	930.50	−0.278	0.781	0.956
Vertigo	864.00	−0.869	0.385	0.956
Orientation problems	883.00	−0.690	0.490	0.956
Stomach discomfort	806.50	−1.356	0.175	0.956

Subjective experience was assessed in 14 domains by means of a VAS. Adjusted *p*-values are calculated according to the Benjamini-Hochberg correction for multiple comparison.

### 3.2 Protocol guideline

**Figure 7:**
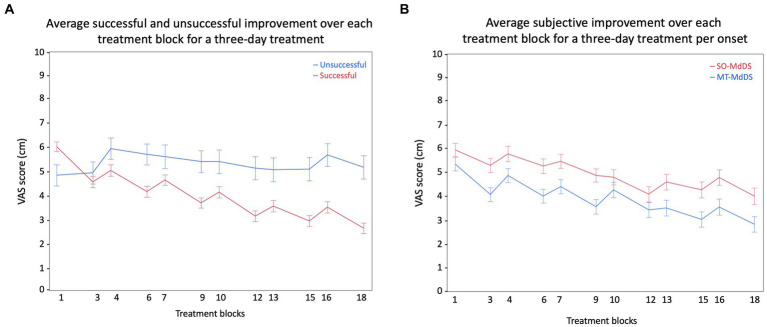
Visualization of the difference in subjective improvement across the pre- and post-session measurements of 3 days. **(A)**, Representation of the difference in successful (*N* = 91) and the unsuccessful (*N* = 39) group regarding subjective improvement. The successful group comprises individuals with a ΔVAS (change in Visual Analog Scale) higher than 1, while the unsuccessful group includes everyone else. **(B)**, The graph depicts a parallel improvement pattern in SO- and MT-MdDS patients, suggesting a comparable response to the standardized OKS treatment paradigm.

Based on subjective improvement, we categorized the ΔVAS (VASpre–VASpost) in three groups; (i) from −10 cm to −1 cm was categorized as unsuccessful or worse, (ii) from −0.99 cm to 0.99 cm was categorized as status quo or no change, and (iii) from 1 cm to 10 cm was categorized as successful or subjective improvement. Using descriptive statistics, based on the ΔVAS categorization of the first treatment, we showed that 8.1% felt worse, 26.3% did not feel any difference or change, and 65.7% subjectively felt better immediately after treatment (Figure 7A). Specifically for the MT-MdDS patients, 4.2% felt worse, 27.7% did not feel any difference or change, and 68.8% subjectively felt better immediately after treatment. Regarding the SO-MdDS patients, 12% felt worse, 26.0% did not feel any difference or change, and 62.0% subjectively felt better immediately after treatment (Figure 7B).

Based on subjective improvement, we aim to provide a protocol guideline. Of the 65.7% of patients who showed significant improvement, most were treated with stripes going left (45%), followed by stripes going right (36.7%), stripes going down (14.3%), and stripes going up (4.1%). Of the patients that showed significant improvement, most were treated for three consecutive days (61%), followed by four consecutive days (14.6%), five consecutive days (13.4%), and two consecutive days (11%). Patients were treated twice a day during a morning session and an afternoon session of two times 4 min of OKS (96.3%), 2 min (2.4%), and 6 min (1.2%).

The most successful protocol, based on our findings, consists of three consecutive days with a morning and afternoon session of two times 4 min OKS, respectively.

## 4 Discussion

### 4.1 Patient characteristics

This retrospective study included a cohort of 50 SO-MdDS patients and 51 MT-MdDS patients. The considerable number of SO-MdDS patients emphasizes the need for a better understanding of the clinical characteristics within this patient subset to facilitate rapid diagnosis and treatment, given that no typical motion trigger can aid in the diagnostic process. Interestingly, a substantial number of foreign patients (59%) sought treatment at the Antwerp clinic after enduring symptoms for an extended period, ranging from 1 month to 21 years (median 3.7 years, MAD 1.8). Many patients had tried all sorts of treatments before, mainly using medication and or vestibular rehabilitation. This persistent pattern highlights the ongoing challenges associated with the recognition of MdDS and that treatment options are limited.

Our study population also emphasizes the significance of understanding the impact of MdDS in women, as 81% of the cohort were female, an occurrence rate that is found in many studies. The average age (SD) of female participants was 45 (15) years old, which is approximately around the age of menopause, aligning with previous research findings (4). As mentioned before, some patients of the SO-MdDS group indicated that they experienced the onset of MdDS after discontinuing contraceptive medication or following birth. Both situations involve significant hormonal shifts, which are also observed during the premenopausal and menopausal phases. The potential role of hormones in the development of MdDS remains an open question, highlighting the need for further research into the influence of hormonal factors on MdDS.

### 4.2 Treatment characteristics

Dr. Dai’s approach for determining the frequency of the head-roll movement in treating MdDS patients was based on the monitored frequencies of patients’ internal perceptions of self-motion using a wrist accelerometer. Patients were instructed to move their wrists in sync with their perceived self-motion frequency. Additionally, posturography measurements were taken to determine this frequency. Furthermore, Dr. Dai used the direction of the nystagmus, recorded through video-oculography, to determine the direction of the stripes. Patients underwent a five-day treatment, consisting of one to eight sessions per day, each lasting 3 to 5 min. Dr. Dai’s treatment approach yielded a success rate of 70% (8). In contrast to their approach, we used a standardized treatment method that was uniform across all patients. We used a fixed head-roll movement frequency of 0.167 Hz, corresponding to the resonance frequency of the vestibular organs (16). The velocity of the OKS stripes was set at 10° per second, and our patients were mostly treated for three consecutive days twice a day, with two times 4 min OKS and head-roll. The direction of the stripes was kept constant throughout the treatment period, unless no clear improvement was observed after several sessions. The current study was based on the protocol performed by Mucci and colleagues reporting that there were no significant postural improvement when adding a fourth and fifth day of treatment, implying patients did not experience any benefit from additional treatment days (14).

Based on other studies, we used the VAS score and a symptom-specific questionnaire for the subjective assessment of the treatment’s impact (14, 20, 21). As objective outcome measures, we utilized posturography, specifically assessing CEA, AUC-ML, and AUC-AP. Posturography has already been proven to be a valuable tool for the quantification of balance, as it objectively measures postural sway and the velocity rate of sways in vestibular patients, representing postural imbalance (14, 22–24). Nevertheless, it is important to recognize that postural control may be affected by additional factors that should be considered when analyzing posturography outcomes. These factors include inter- and intra-subject variability, the presence of single or multiple disease entities, disease severity, habituation, ‘first trial’ effects, and the impact of cognitive and emotional factors (25).

We observed both subjective and objective improvement after the treatment. Considering the VAS improvement, our findings indicated an overall success rate of 66%, slightly lower than Dr. Dai’s 70% (8). It is important to consider several differences between both study populations, such as the inclusion of both MT-MdDS and SO-MdDS patients in our study population, and the number of treatment sessions. Even though our success rate appears to be slightly lower than Dr. Dai’s, our more standardized protocol yields comparable benefits and enhances the level of standardization.

Furthermore, we showed an effect of re-treatment on the improvement in VAS score pre- to post-treatment. Note, however, that the number of patients who received multiple treatments was considerably smaller. Additionally, the effect of treatment was only visible at the level of subjective improvement and not for the posturography outcome measures (CEA, AUC-ML, and AUC-AP). The difference between this objective and subjective finding can be assigned to the fact that self-reported cognitive functioning cannot serve as an adequate substitute for performance-based cognitive functions (26). This can be attributed, for instance, to the fact that patients perceive internal oscillations differently. This can result in varying VAS scores, even though patients may experience the same level of symptom severity. However, it is important to emphasize that subjective improvement holds equal significance compared to posturography. Subjective improvement is invaluable as it directly mirrors the patient’s perception of their own well-being. Both the VAS and posturography are distinct measurements, with neither outweighing the other in importance. Hence, the advantage of additional treatment sessions over the initial treatment remains uncertain, as no significant effects were observed for treatment (from the first to the fourth session) on the objective outcome measures.

Our results showed significant correlations between all subjective (VAS score) and objective outcome measures (CEA, AUC-ML, AUC-AP). This shows that after the treatment session, the patients experienced a subjective improvement which could be objectively quantified by the posturography outcome measures.

After the treatment sessions, there was a significant improvement observed in all 14 symptoms assessed by the MdDS symptom specific questionnaire. Before treatment, an extensive number of patients indicated they felt less lively, hindered in interactions with friends or family, felt down in the dumps and gloomy, tired, unhappy, and scared their health might deteriorate even more. These results show the extensive impact of MdDS, not only on physical health but also on mental well-being and QoL. Based on symptom improvement post-treatment, this shows that the OKS treatment is beneficial in increasing the QoL and mental well-being. A previous sham-controlled study demonstrated no placebo effect of OKS, suggesting that the improvement in QoL is not the result of merely receiving a diagnosis and treatment, but of the actual treatment targeting the primary symptom of self-motion perception (14). Nonetheless, the improvements in QoL likely arise from other factors as well besides the actual treatment, explaining some of the observed differences between subjective feelings and actual stability. This implies that a definitive assessment of the effectiveness of treatment modalities can only be provided when we gain a deeper understanding of the underlying pathophysiological mechanisms of MdDS (27, 28).

In terms of the onset type, MT- versus SO-MdDS, our analysis revealed no significant difference in success rates, VAS score, symptom improvement, and posturography outcome measures. These results are contradictory to prior research, which suggested that the OKS treatment might be less effective for SO-MdDS patients (14, 22, 29). This result also suggests that SO-MdDS has a larger overlap with MT-MdDS than it has with Persistent Perceptual Postural Dizziness (PPPD), a diagnostic category that some clinicians use as more appropriate for SO-MdDS patients. It is likely that the equally successful outcome from OKS treatment for MT- and SO-MdDS patients reflects their shared characteristic of temporary symptom relief when (re-)exposed to passive motion. Considering our treatment outcomes, we advise to use a standardized approach for treating all MdDS patients, regardless of their onset, particularly for scientific research purposes. We recommend a treatment regime of three consecutive days, with two blocks of 4 min in the morning and afternoon. Between the morning and afternoon sessions, we recommend that patients stay active, take walks, and engage in activities, e.g., ball sports, to stimulate the vestibular system with natural movements. The OKS stripes are set to move at a speed of 10°/sec, and a fixed frequency of 0.167 Hz for rolling the head from the left to the right shoulder should be used. After three treatment days, clinicians may opt for customized treatment sessions, for example by adjusting the direction of stripes if needed in accordance with the patient’s internal perception of oscillation or with the deviation of the Fukuda stepping test.

### 4.3 Future challenges

One of the primary challenges we face is the limited understanding of the underlying mechanisms that play a role in both MT-MdDS and SO-MdDS subtypes. In a study conducted by Browne and colleagues, it was revealed that MT-MdDS patients use on average 3.9 different treatment strategies, while SO-MdDS patients use 4.5 strategies. These strategies include various interventions, such as antidepressants, vitamin supplements, physiotherapy, and osteopathy (27). The use of different treatment strategies may be attributed to the complexities associated with diagnosing MdDS patients, particularly those with the SO subtype. However, our research findings indicate that there are no differences in the perceived benefits of our treatment strategy between SO-MdDS and MT-MdDS patients. This observation suggests that although these subtypes have difference onset causes, they may share a common underlying pathophysiological mechanism (27).

MdDS symptoms can vary between patients, but also within the same individual, fluctuating from day to day or following individual treatment sessions. The success of the treatment, however, is dependent on the correct intervention at the right time, which demonstrates the complexity of treating MdDS patients (22). Another challenge in the treatment of MdDS patients remains the possible relapse or aggravation of symptoms after additional travel. While one study suggests that continued travel by boat or plane does not extend the duration of MdDS episodes (30), it emphasizes the importance of patients avoiding passive transportation within the initial 2 weeks after the treatment to maintain the treatment’s improvement (23).

## 5 Limitations

The assessment of symptom severity was conducted using the VAS scale. It is important to note that this scale lacks standardization and may demonstrate variations among patients, influenced by factors like age, past experiences, and sex. This makes the calculation of averages between subjects complicated, while the improvement within the same patients is more robust. In addition to the VAS, we used the MdDS symptoms specific questionnaire to obtain a more comprehensive insight into MdDS-related aspects. Nonetheless, the introduction of a more comprehensive and personalized grading system could help mitigate interindividual variations in reporting symptom severity.

While posturography measurements effectively capture swaying and rocking sensations, the bobbing sensation (i.e., up and down movement) is less clear in posturography analysis, while some patients specifically report this type of self-motion perception. Further research is needed to develop improved assessment methods for collecting more statistically reliable data from patients experiencing the bobbing sensation. Additionally, investigations should be conducted to ascertain whether treatment involving stripes in a downward motion should include an up/down head-pitch movement, in contrast to the current side-to-side approach.

This retrospective study solely examined the immediate impact of the OKS treatment and did not incorporate a follow-up phase. Prior research has incorporated follow-up phases utilizing questionnaires; however, the inclusion of an additional objective measure would provide a more comprehensive assessment of postural improvements following treatment (14, 22, 23, 29).

## 6 Conclusion

Based on Dr. Dai’s OKS paradigm, we adopted a more standardized treatment approach applicable to all MdDS patients, either motion triggered or those of the type spontaneous or other onset. Our treatment typically consisted of 3 days, with morning and afternoon sessions lasting two times 4 min each. The OKS was presented at a rate of 10°/sec, while the head was rolled side-to-side at 0.167 Hz. Both MT and SO MdDS patient groups reported subjective and objective improvements following the treatment sessions. The overall success rate for MdDS treatment was 64.1%, with no statistically significant difference between MT-MdDS patients (64.2%) and SO-MdDS patients (63.3%), suggesting similar underlying mechanisms between both subgroups. Our treatment protocol could serve as a standardized approach or guideline for treating both types of MdDS patients.

## Data availability statement

The original contributions presented in the study are included in the article/supplementary material, further inquiries can be directed to the corresponding author.

## Ethics statement

The studies involving humans were approved by Commissie Medische Ethiek GZA Ziekenhuizen (CME GZA Ziekenhuizen). The studies were conducted in accordance with the local legislation and institutional requirements. Written informed consent for participation was not required from the participants or the participants' legal guardians/next of kin due to the retrospective nature of the study.

## Author contributions

CS: Writing – original draft, Writing – review & editing. SJ: Writing – review & editing. CL: Writing – review & editing. AZ: Writing – review & editing. FW: Writing – review & editing.

## Footnotes

^1^ https://www.colorado.edu/neuromechanics/research/wii-balance-board-project
